# Dr. Corden Thompson on Anatomical Pursuits

**Published:** 1830-04-01

**Authors:** 


**A Letter to the Public, on the Necessity of Anatomical Pursuits, &c. By *Corden Thompson*, M.D. 8vo. pp. 92, 1830.**

Dr. Thompson, in this little pamphlet, which is dedicated to the Marquess of Lansdowne, has evinced great learning and research, together with much keen and critical acumen. But we may remark that, to the medical profession and to the more enlightened and liberal classes of society, such a laboured production, in proof of the utility of dissection, is unnecessary— to the narrow-minded and bigotted, it would carry no conviction, because their hearts are hardened, and their brains are insensible—while to the vulgar, the whole epistle would be Hebrew. But what is more mortifying than all to authorship, such erudite essays, on such a subject, will not be read, by any member of society, in or out of Parliament—not, at all events, by one in a million, which would make about 27 readers in England, Ireland, and Scotland. We know indeed that Dr. Thompson will be able, in the course of 1830, to produce more than 27 proofs of the incorrectness of this assertion, in the shape of letters from the Marquess of Lansdowne, and many others, full of compliments, and full of asseverations as to the pleasure received during the perusal of the letter; and we are really sorry to disturb the pleasure which the receipt of congratulatory letters is calculated to convey. But some knowledge of the world, and some intercourse with its motley inhabitants, have taught us a few secrets on these points. The dissection of human bodies is dirty work at the best, and nothing but the love of *anatomical* science can ever render it bearable, much less pleasing. This love will never be generally diffused among mankind at large, even by the eloquent harrangues and lectures of Dr. Birckbeck. They are so much occupied with the things of this world—eating, drinking, and money-making—and so enamoured with the prospect of their carcases taking on angelic forms, at the resurrection, that they never will bear the idea of dismemberment by the uncleanly hands of the anatomist. The following extract from the letter before us affords a tolerably convincing proof of the justness of these observations.

"What, then, must we candidly think of those legislators who have joined the popular outcry against dissection? Do they, with the vulgar, suppose that it consists simply in cutting and hewing to pieces? Do they never think of it except in connexion with a bloody knife? It would really seem so. The reverend gentlemen in lawn appear not to have forgotten the epithet applied to Herophilus by Tertullian.* To account for their conduct, we are compelled to suppose, that they regard practical anatomy as a sort of impious mangling of the dead; a species of butchery which, possibly, with a pious horror, they wish again to see *banished from our universities*. A very fine specimen of this sort of feeling has lately been displayed by a *Divine* of the Scotch Church. Mr. Pollok,

---

* "Herophilus ille, medicus aut *Lanius*, qui Sexcentos exsecuit ut naturam scrutaretur." Ed.

in his operose production on the 'Course of Time,' speaking of the resurrection at the last day, thus introduces the anatomist :—

> "And as the anatomist, with all his *band*
> Of *rude disciples*, o'er the subject hung,
> And *impolitely (!) hewed his way*, through bones
> And muscles of the *sacred* human form,
> *Exposing barbarously to wanton gaze,*
> The *mysteries of nature*, joint embraced
> His kindred joint, the *wounded flesh* grew up,
> And suddenly the *injured* man awoke,
> Among their hands, and stood array'd complete
> In immortality—*forgiving scarce*
> The *insult* offered to *his clay* in death."

"The preceding is *one* only of the *numerous* instances of wretched taste and ponderose diction with which this admired (!!!) writer abounds. But the taste of the multitude consists in having *no taste* at all. There never was, we will venture to affirm, a more complete example of the *Bathos* in writing than the one now cited. 'The anatomist with his *rude band impolitely hewing his way through bones and muscles*:' aye! bones and muscles of the *sacred* human form! *Hic labor, hoc opus est!* But to worms and other vermin it is quite a delightful and recreating amusement to find their way through these parts, *sacred* as they are in the poet's eye. And, then, to *expose barbarously to wanton gaze the mysteries of nature!* Horror of horrors! Execrable wretches! What! strip nature naked, and expose her to the wanton gaze of a rude band of dissectors? Proh pudorem; And again; only think, the poor *injured* man, after the resurrection, scarcely can *forgive* the *insult* offered to *his clay* in death! What an elevated and sublime idea! How truly Christian like!! Now, suppose he had found a thousand revelling worms at work; or a huge fish tearing off a limb; or a wild beast picking a bone;—would he have forgiven these creatures? And, after all, we dare say the numerous readers of this Scotch Milton (!!!) find a *comfortable* strengthening of their prejudices in perusing this pitiable nonsense; for pitiable, indeed it is, when we recollect the concise and animated language of Paul. Yet, be it remembered, this linsy-woolsey stuff is from the pen of a divine in the nineteenth century; from one who talks about the 'unfallen, holy, religious sea!' How forcibly does it remind us of *Merops* in Lessing's fables. The reader will excuse our translating it for him :—

"I wish to ask you a question," said a young eagle to a very sage and learned owl. "There is a bird, they say, called Merops, which, when it rises in the air, flies with the tail foremost, and the head towards the earth. Is this true?" "Nonsense, child!" answered the owl; "this is a mere silly fiction of *man*. Perhaps *he is himself such a kind of Merops*, for he is always *trying to fly up to heaven, but never by any chance loses sight of earth!*" 51.

If reverend divines will thus aid in fostering the prejudices of mankind against the practical study of anatomy, what can we expect from those who have no liberal education?

But medical science, or, at all events, the major mass of its professors, is menaced with a greater evil than the difficulties attendant on dissection. Bodies will be procured, in greater or less abundance, by money, whenever they are wanted. We have the authority of Sir Astley Cooper, before a Committee of the House of Commons, that any man's body may be brought into the dissecting-room, by that potent agent gold. There is a far worse prejudice against the profession rapidly engendering in the public mind, which their passions have not, for some time past, permitted them to perceive, but which their pockets, and their sober judgment are now beginning to feel. The scandalous and abusive language introduced into medical politics, and carefully doled out by Reporters in the daily newspapers, has created an unfavourable impression in the public mind against the whole profession of medicine! A general distrust, we might also say *contempt* prevails, against the medical character, and, if things went on as they have lately done, we should not be surprised if the character of medical practitioners in this country fell to the level of that in Spain—where a barber would consider himself disgraced, if his daughter married the son of a physician! We happen to know something of the prevailing sentiments among the non-professional as well as the professional public; and we venture to affirm that confidence in the integrity of the profession is rapidly waning—and the consequence is, and will be, that all classes of society will avoid, as much as possible, communication with medical practitioners, while those in affluent circumstances will only have recourse to men whose reputation and character place them beyond the reach of suspicion.

How can it be otherwise?—Custom has introduced two or three classes or distinctions in the practice of medicine. Modern medical tactics have introduced the plan of setting one class in opposition to the other, and of employing the most disgraceful and Billingsgate language in these collisions! The consequence is what we have stated above—and the banks of Swan-river will ultimately swarm with medical practitioners unable to find the means of subsistence in England.

We regret that Dr. Thompson did not adopt a more concise, popular, and persuasive strain in his letter. He is evidently possessed of talents, learning, and acumen, which might have been turned to excellent account, in a less laboured and erudite epistle.

